# Modelling and Characterization of Effective Thermal Conductivity of Single Hollow Glass Microsphere and Its Powder

**DOI:** 10.3390/ma11010133

**Published:** 2018-01-14

**Authors:** Bing Liu, Hui Wang, Qing-Hua Qin

**Affiliations:** 1 College of Civil Engineering and Architecture, Henan University of Technology, Zhengzhou 450001, China; bingliu@stu.haut.edu.cn; 2 State Key Laboratory of Structural Analysis for Industrial Equipment, Dalian University of Technology, Dalian 116024, China; 3 Research School of Engineering, Australian National University, Canberra, ACT 2600, Australia

**Keywords:** hollow glass microsphere, thermal conductivity, transient plane source technique, hierarchical computation

## Abstract

Tiny hollow glass microsphere (HGM) can be applied for designing new light-weighted and thermal-insulated composites as high strength core, owing to its hollow structure. However, little work has been found for studying its own overall thermal conductivity independent of any matrix, which generally cannot be measured or evaluated directly. In this study, the overall thermal conductivity of HGM is investigated experimentally and numerically. The experimental investigation of thermal conductivity of HGM powder is performed by the transient plane source (TPS) technique to provide a reference to numerical results, which are obtained by a developed three-dimensional two-step hierarchical computational method. In the present method, three heterogeneous HGM stacking elements representing different distributions of HGMs in the powder are assumed. Each stacking element and its equivalent homogeneous solid counterpart are, respectively, embedded into a fictitious matrix material as fillers to form two equivalent composite systems at different levels, and then the overall thermal conductivity of each stacking element can be numerically determined through the equivalence of the two systems. The comparison of experimental and computational results indicates the present computational modeling can be used for effectively predicting the overall thermal conductivity of single HGM and its powder in a flexible way. Besides, it is necessary to note that the influence of thermal interfacial resistance cannot be removed from the experimental results in the TPS measurement.

## 1. Introduction

Inorganic hollow glass microsphere (HGM) is a white tiny bubble with the micron-scaled diameter and wall thickness. Such hollow structure and fine spherical shape makes HGM have some distinctive properties, such as high compressive strength, low density, low water absorption, low heat conduction, and high chemical resistance [1,2,3], which are important to develop new structural materials, such as multilayer sandwich composites [1,4], syntactic foams [5,6,7,8,9,10,11], and lightweight concretes [12,13,14,15], when compared to conventional organic fillers [16,17], for civil engineering, deep-sea exploration, and hydrogen storage. Besides, due to its low thermal conductivity, HGM can be applied as an insulating material to meet the increasing requirement of energy saving. For example, Li et al. explored the thermal insulation performance of HGM and proved that the heat transfer in HGM is dominated by conduction [18]. Zhu et al. studied the thermal properties of low-density polyethylene (LDPE) composites filled with HGMs [19]. Recently, their study was extended to LDPE/epoxy blend filled with HGMs and nitride particles [20]. The works above revealed that the effective thermal conductivity of composites decreases with the increase of volume content of HGM. This can be attributed to the existence of interior gas core of HGM. However, up to date, most existing studies of HGM-based composites focus on the development of new composites and little work has been seen for the prediction of thermal conductivity of HGM itself independent of any matrix [18], although it is a key factor for a fully conscious design of insulating materials. In order to apply the HGM better in the insulation field, it is necessary to further understand the effects of its structure and physical parameters on its insulation performance. Moreover, owing to the limitation of its spherical shape, it is inconvenient to directly measure the thermal conductivity of HGM.

This work focuses on the determination of thermal conductivity of HGM aggregates experimentally and numerically. Firstly, the thermal conductivity of the HGM powder is measured by means of the transient plane source (TPS) method. To alleviate the influence of non-homogeneity of HGMs in the powder as much as possible, multiple measurements are performed for different stacks of the HGMs. Subsequently, the thermal conductivities of the three different HGM stacking elements that are assumed to approximately represent the distributions of HGM in the powder are numerically determined by the present three-dimensional (3D) two-step hierarchical computational method, which has been successfully developed for the two-dimensional natural fiber bundle [21]. The comparison of the experimental results and the numerical simulation validates the present modelling.

The paper is organized as follows. Section 2 describes the geometrical and structural features of HGMs. The TPS theory is briefly introduced and the related experimental program is described in Section 3. Then the computational strategy developed in this work is presented in Section 4 and related results are discussed in the same section. Finally, some conclusions are summarized in Section 5.

## 2. Hollow Glass Microspheres

**Figure 1:**
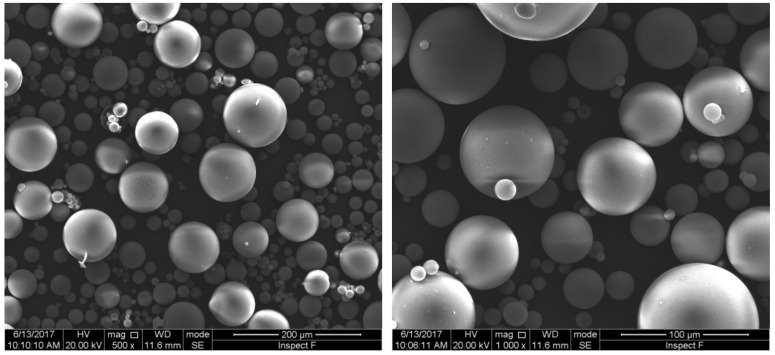
Scanning electron microscope (SEM) images of hollow glass microspheres.

**Figure 2:**
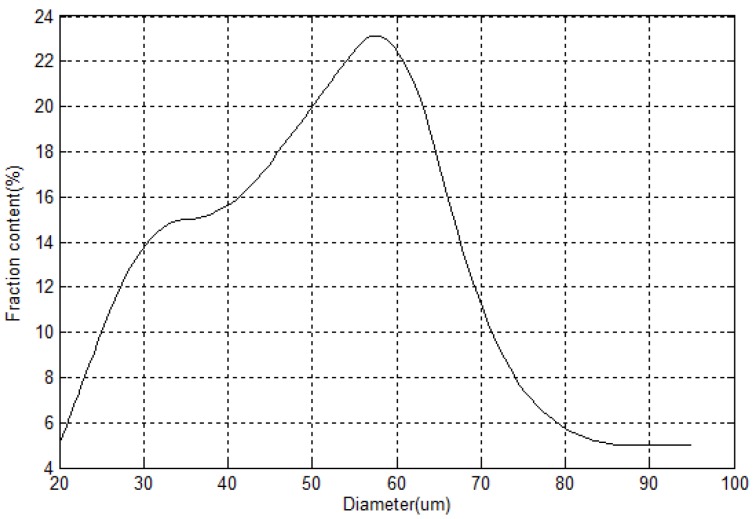
Distribution of diameters of microspheres.

**Figure 3:**
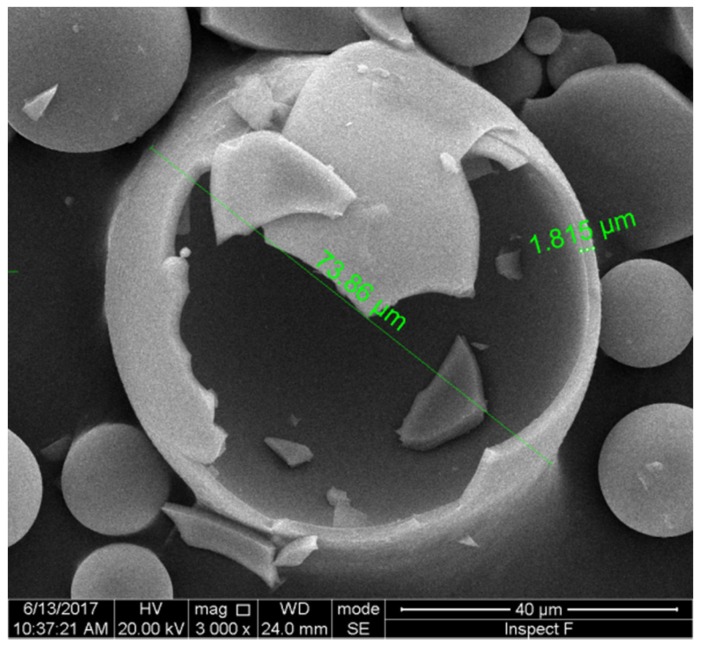
SEM image of cracked hollow glass microspheres.

The manufacture of HGM involves complex hydrodynamic and chemical processes; it is impossible to make microspheres with identical diameter and wall thickness. In order to understand the insulation behavior of HGM better, which is closely related to its microsphere size and microstructure, typical scanning electron microscope (SEM) images of HGM involving full view and cross section are provided here. The observing experiment is conducted by using the FEI Quanta 250 FEG machine at Henan University of Technology. Figure 1 displays two SEM images of HGMs in different scales, which were fabricated by Sinosteel Maanshan New Material Technology Co., Ltd (Maanshan, China, www.glass-bubble.com). From Figure 1, it is clearly seen that the tiny bubbles are in perfect spherical shape, but their diameters are distributed in a certain range. Therefore, it is necessary to perform statistics of microsphere’s dimension. Figure 2 demonstrates the distribution of diameter of microspheres, from which it is observed that the size of microsphere mainly locates in the interval [30 μm, 70 μm]. Besides, the wall thickness of hollow microspheres is another interesting geometric factor. Figure 3 shows the cross section of a typical cracked microsphere. The clear hollow structure and homogeneous wall thickness is observed from this figure. Based on the measured value of each cracked microsphere, it is found that the wall thickness of the microspheres changes from 1.2 to 2.2 microns. However, it is interesting that the void content to the microsphere keeps nearly 85% unchanged, although the wall thickness of HGM increases with the increase of its diameter.

**Figure 4:**
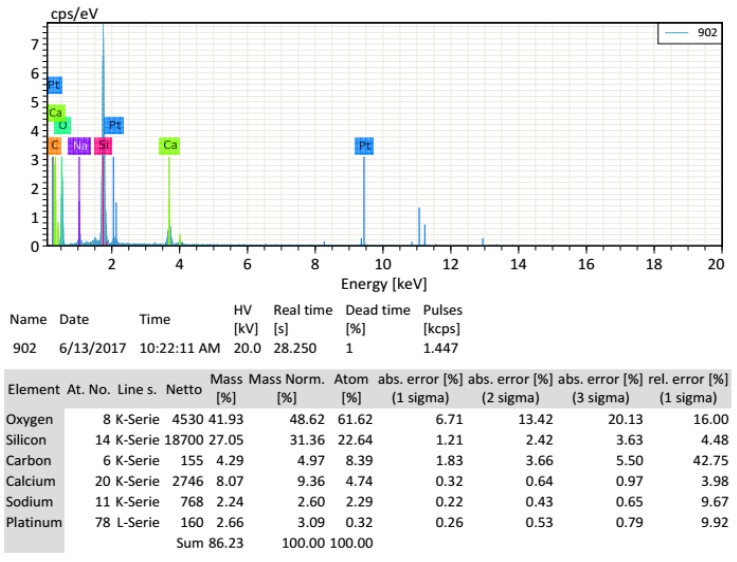
Energy spectrum of solid wall of hollow glass microsphere (HGM).

In addition to the dimensions of microsphere, another important issue is to determine the chemical composition of its solid wall. To do so, the energy spectrum of solid wall of HGM is measured in the same equipment FEI Quanta 250 FEG. Figure 4 indicates the wall of HGM is mainly composed of about 80% of SiO_2_ and 20% CaO.

## 3. Experiment

### 3.1. Introduction of the TPS Method

**Figure 5:**
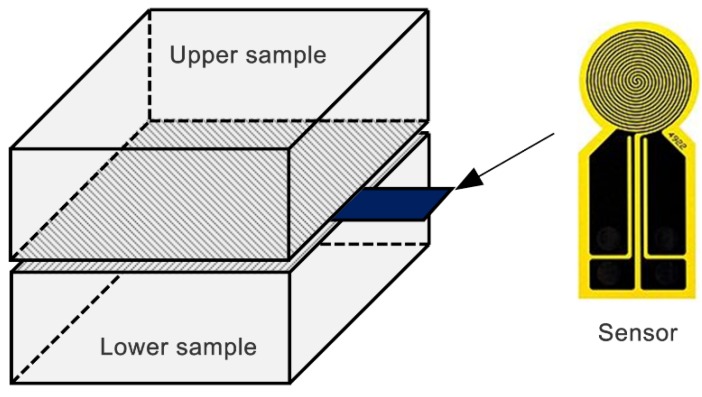
Principal experimental setup for the transient plane source (TPS) method and the sensor shape.

In this study, the TPS method proposed by Gustafsson [22] is used to measure the thermal conductivity of HGM powders. It has become an ISO standard (ISO22007-2) for rapidly and precisely measuring thermal transport properties of bulk materials [22,23]. The principle of the TPS method is based on the introduction of plane sensor element that acts both as dynamic temperature sensor and heat source. This plane sensor usually consists of a double spiral heating element made of thin pure nickel foil (about 10 μm) and two thin insulating layers made of Kapton (about 70 μm). In the practical measurement, this plane sensor should be placed between two identical samples with both sensor faces being in contact with the two sample surfaces, as depicted in Figure 5.

When the plane sensor element is electrically heated, the value of its electric resistance R(t) in the sensor can be presented as a function in terms of the average temperature increase ΔT(t) of the sensor element by the following expression

(1) $$R(t)=R_{0}[1+\alpha \Delta T(t)]$$

where t is the test time, $R_{0}$ is the initial electric resistance of the sensor, and α is the temperature coefficient of resistance of the nickel.

With the assumption that the sensor acts as a number of concentric and equally spaced ring sources, the average temperature increase in the sensor can be conveniently written as [22,23]

(2) $$\Delta T(t)=\frac{P_{0}}{\pi^{3/2}ak}f(\tau )$$

where $P_{0}$ is the power output of the sensor, a is the radius of the sensor, k is the thermal conductivity of the test sample, $\tau =\sqrt{t/\Theta }$ is the dimensionless time, $\Theta =a^{2}/\kappa$ is the characteristic time and κ is the thermal diffusivity of sample material, f(τ) is dimensionless time function representing the energy accumulating effect during the time [0,*τ*], and it is related to the number of ring sources in the sensor. Its expression can be found in [22].

From Equation (2), it is found that the average temperature increase ΔT(t) in the sensor increases linearly with respect to the dimensionless time function f(τ). Thus when measuring thermal conductivity of a sample, the curve of average temperature increase vs heating time in the sensor under given input power and heating time can be plotted based on Equation (1). Then, the approximated line for the relationship (2) between ΔT(t) and f(τ) can be obtained by fitting experimental data and the slope of the line can be used to determine the thermal conductivity of sample, according to Equation (2).

In comparison with the conventional guarded hot plate method, the TPS method is a transient method that has the advantages of simplicity and efficiency in experiment, and it can accommodate a wide thermal conductivity range (i.e., 0.005–300 W/(mK)) and is able of measuring various kinds of materials, including solids, liquid, powder, and thin films.

However, it is necessary to point out that, limited to the surface roughness of samples, the actual contact area is usually significantly smaller than the apparent contact area between bodies in contact, thus a thermal interfacial resistance is unavoidably caused, which may affect the ability to conduct heat between them [24,25,26,27,28,29,30]. Therefore, the thermal interfacial resistance can be viewed as an inherent property, independent of the measurement method, such as the TPS used in the study. As a result, the thermal interfacial resistance between the TPS sensor and the samples are clearly included in the measured values of thermal conductivities in this paper [24,26,27,28,29].

### 3.2. Experimental Program

**Figure 6:**
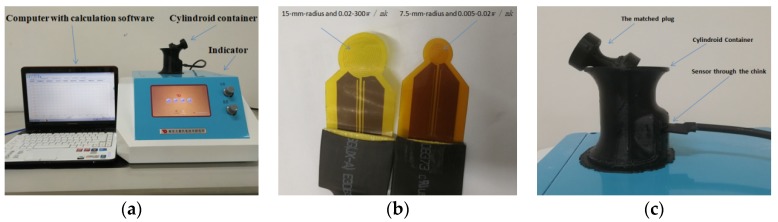
The TPS equipment for powder measurement (**a**) Indicator; (**b**) two sensors; (**c**) cylindroid container.

In the experiment, the thermal conductivity is measured by DZDR-S Thermal Constants Analyzer based on the TPS technique, provided by Dazhan Institute of Electromechanical Technology, Nanjing, China. As shown in Figure 6a, the equipment typically consists of an indicator that is used for adjusting the voltage, a computer with testing software and two different sensors (see Figure 6b), which can be used in different measuring ranges of thermal conductivity, 0.005–0.02 W/(mK) and 0.02–300 W/(mK), respectively. The radii of the nickel foil in the two sensors are 7.5 mm and 15 mm, respectively. The thermal conductivity of microsphere powder can be measured using the two sensors. A black special container shown in Figure 6c is used to provide an approximate closed and insulated environment for the powder in it. Besides, as seen in Figure 6c, there is a thin slot in the wall of the container to let the sensor pass through it and contact to the powder. A relatively small constant force required for the contact method, i.e., 10 N, is applied via a matched weighted plug provided by the manufacturer to make the powder more compact and simultaneously to avoid fracture of the microspheres [31].

When considering the size difference of microspheres, the HGM powder is poured into the container four times for adjusting the local distribution of HGMs around the sensor element, which resulted in four testing groups referred as Group A, B, C, and D, respectively. For each group, heating powers (in 0.061 W, 0.071 W, and 0.081 W) are applied, respectively, and the corresponding temperature increases are recorded. Table 1 shows the results of the multiple measurements, the averaged value of thermal conductivity of the HGM powder is 0.1014 W/(mK).

## 4. Computational Model and Results

### 4.1. Three Different HGM Stacking Elements

**Figure 7:**
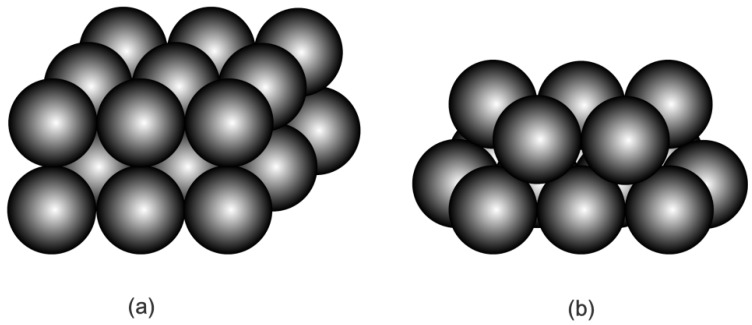
Assumed configurations of HGMs in the powder: (**a**) the periodic cubic close-packing and (**b**) the periodic hexagonal close-packing.

**Figure 8:**
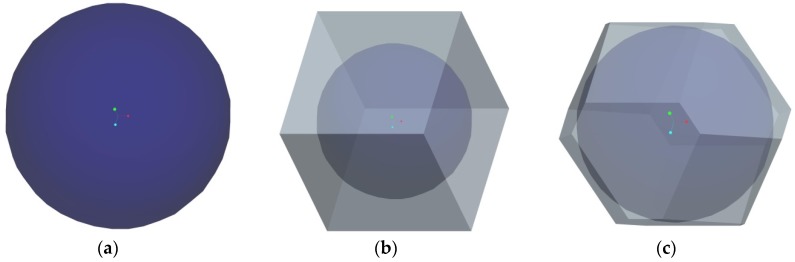
The corresponding HGM stacking elements: (**a**) the single HGM stacking element; (**b**) the cubic HGM stacking element and (**c**) the truncated octahedron HGM stacking element.

It is assumed that HGMs in the powder are mainly distributed in three different configurations. The first configuration is built by thoroughly neglecting air voids in the powder, so that a single HGM can be considered as stacking element for such an extreme case. The second configuration is the cubic close-packing of HGMs in the powder (see Figure 7a) so that a cubic stacking element, including a HGM and surrounding air void can be taken from the powder for consideration. The third configuration is the hexagonal close-packing of HGMs in the powder (see Figure 7b), so that a truncated octahedron stacking element including a HGM and surrounding air void is considered as representative unit cell of this kind powder. The corresponding HGM stacking elements for the three distribution assumptions are shown in Figure 8. Different to the single HGM stacking element, both the cubic HGM stacking element and the truncated octahedron HGM stacking element introduce external air voids surrounding the centered HGM. Accordingly, the external air volume fraction to the stacking element is 0% for the single HGM stacking element, 47.7% for the cubic HGM stacking element and 21.9% for the truncated octahedron HGM stacking element, respectively. Our aim is to determine their thermal conductivity in this section.

### 4.2. 3D Two-Step Hierarchical Computational Method

**Figure 9:**
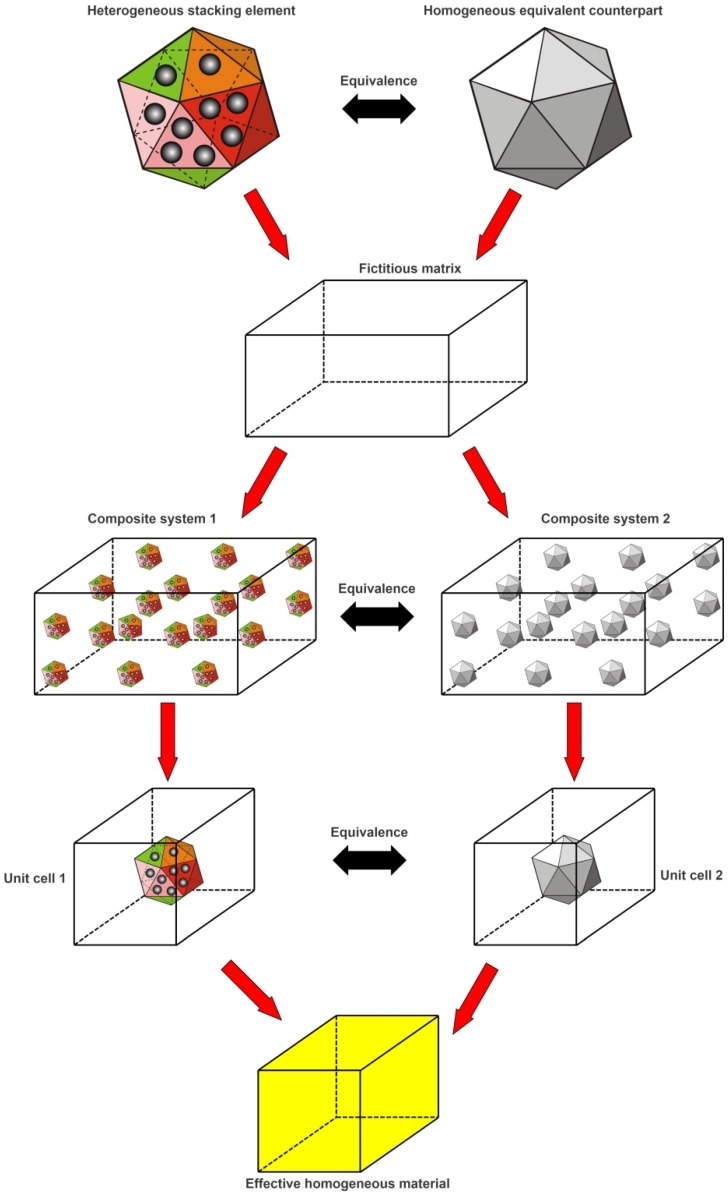
Schematic diagram of basic procedure of the present method for arbitrary stacking element.

Evidently, the three stacking elements listed above typically have irregular shapes and heterogeneous microstructures, and thus it is difficult to directly measure the thermal conductivity of them. But, we can do this through numerical approaches. There are many models for evaluating effective materials properties of heterogeneous materials [32,33,34]. In this paper, a three-dimensional two-step hierarchical computational method is proposed to predict the effective thermal conductivity of the three particular stacking elements described in Section 4.1, and the basic procedure of it is described in Figure 9 for a general heterogeneous stacking element. In Figure 9, the two equivalent fictitious composite systems (system 1 and system 2) are respectively established by periodically embedding the interesting heterogeneous stacking element and its homogeneous equivalent solid counterpart as fillers into the same fictitious matrix material with same filler volume fraction. The stacking element and its equivalent counterpart has the same outermost shape and dimension. If the periodic cubic pattern is assumed for the distribution of the interesting stacking element and its equivalent counterpart in the fictitious matrix material, two corresponding cubic composite unit cells (Unit cell 1 and Unit cell 2) can be, respectively, taken out from the two fictitious composite systems for our study. The Unit cell models are based on the most basic and smallest cell that can be repeated periodically to form the entire composite medium, so that the physical properties of the unit cell represent the properties of the entire composite material [24,28,35,36]. In the present method, the two unit cells can be modelled by the standard finite elements to obtain the temperature and heat flux distributions in them. Finally, the equivalency of the fictitious composite systems can be used to bridge the stacking element and its equivalent counterpart to determine the value of the whole thermal conductivity of the complex filler.

In order to illustrate the detailed procedure of the present method, we take the stacking element (a) in Figure 8 as an example, and the stacking elements (b) and (c) can be similarly treated. Table 2 shows the geometrical dimensions of HGM and the thermal properties of involved material phases in the simulation. It is worth noting that the wall thickness in Table 2 is evaluated by 85% of the void content to the microsphere, as discussed in Section 2. Besides, the thermal conductivity of the solid wall composed of CaO-SiO_2_ system is approximately given through the rule of mixture as 1.03 W/(mK) [37], which is close to that of glass [38]. The thermal conductivity of matrix material is assumed to be 0.93 W/(mK), unless specially stated. In the analysis, the fictitious matrix material is just introduced in the computational method to form new composite systems, thus the predicting results of the microsphere should theoretically be independent of the choice of matrix material. Additionally, it is assumed that each material phase is isotropic and homogeneous.

#### 4.2.1. The Composite System with Actual Filler

**Figure 10:**
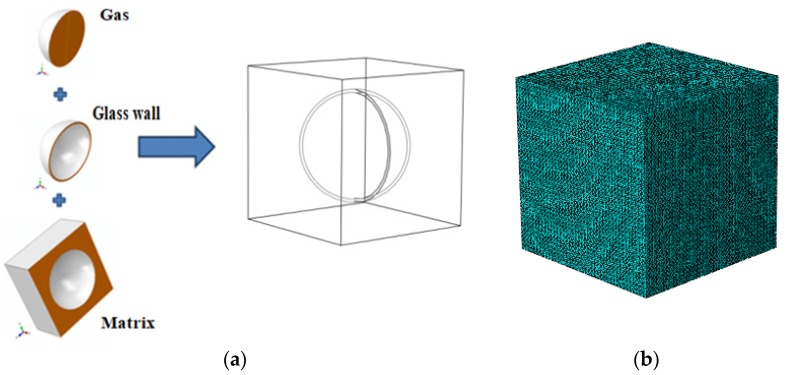
(**a**) The three-phase composite unit cell 1 and (**b**) the related mesh division.

Under the assumption of periodic cubic distribution of the HGM stacking element shown in Figure 8a in the fictitious matrix, a cubic unit cell can be chosen from the three-phase composite system 1 for simulation, as shown in Figure 10. Let *L*, *R* and *t* represent the side length of unit cell, the outer radius of the HGM, and the thickness of its solid wall, respectively, then the volume fraction of the HGM filler to the composite cell can be written as

(3) $$f_{HGM}=\frac{4\pi R^{3}}{3L^{3}}$$

In practical analysis, it is assumed that the outmost radius *R* of the HGM keeps unchanged and the side length *L* of the unit cell can be calculated from Equation (3), with a given value of the microsphere volume fraction to the composite unit cell, i.e., 10%, 20%, 30% and 40%. Figure 10a shows the established 3D unit cell model with 20% HGM volume fraction and Figure 10b displays the finite element discretization with a total of 156,772 elements (DC3D10) and 228,117 nodes that are generated by ABAQUS. In order to achieve accurate and convergent results, a relatively high mesh density is employed here, such that the maximum relative difference in the predicted thermal conductivity between two different meshing schemes is less than a specified tolerance, i.e., 0.1%.

#### 4.2.2. The Composite System with Equivalent Filler

**Figure 11:**
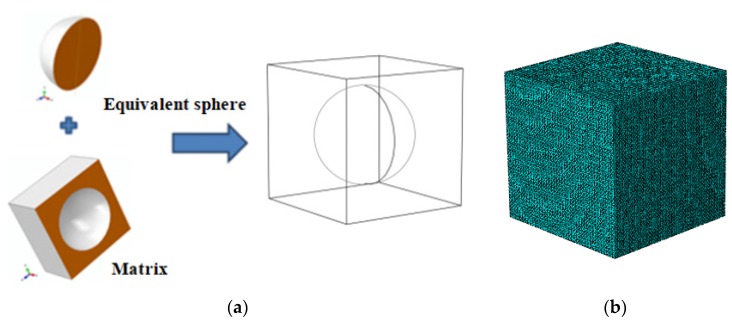
(**a**) The two-phase composite unit cell 2 and (**b**) the related mesh division.

If the HGM filler in the composite system 1 described above is replaced with its equivalent homogeneous solid spherical counterpart with same radius *R*, a two-phase composite system 2 can be naturally formed. Figure 11 shows the unit cell of the two-phase composite system 2 and the corresponding finite element mesh is generated with 153,505 finite elements (DC3D10) and 223,744 nodes.

#### 4.2.3. Basic Heat Transfer in the Two Composite Systems

To evaluate the effective thermal conductivity of the composite systems described above, the heat transfer behavior in them is fully accounted for. By neglecting the effects of heat convection and radiation in tiny particles [39,40], we only consider heat conduction in different material phases. In such a case, the heat balance in the three-dimensional composite unit cell is governed by [40]

(4) $$\nabla^{2}T_{i}(x,y,z)=0,\;i=1,2,\cdots ,n$$

where $T_{i}$ is the temperature of the *i*th material phase and *n* is the number of material phases. $\nabla^{2}=\nabla \cdot \nabla$ and ∇ is the standard del operator in the three-dimensional Cartesian coordinate system (x,y,z).

The constitutive equation describing the relation of the heat flux vector $q_{i}$ and the temperature gradient $\nabla T_{i}$ can be written by the Fourier’s law as [41]

(5) $$q_{i}(x,y,z)=-k_{i}\nabla T_{i}(x,y,z),\;i=1,2,\cdots ,n$$

where is the thermal conductivity of the *i*th material phase.

Besides, the continuous conditions at the interfaces of two adjacent material phases can be given by

(6) $$\begin{array}{l} T_{i}(x,y,z)=T_{j}(x,y,z)\\ k_{i}\frac{\partial T_{i}(x,y,z)}{\partial n}=k_{j}\frac{\partial T_{j}(x,y,z)}{\partial n} \end{array}$$

where the subscripts i and j represent the numberings of two adjacent material phases, respectively, and *n* is the unit normal to the interface.

**Figure 12:**
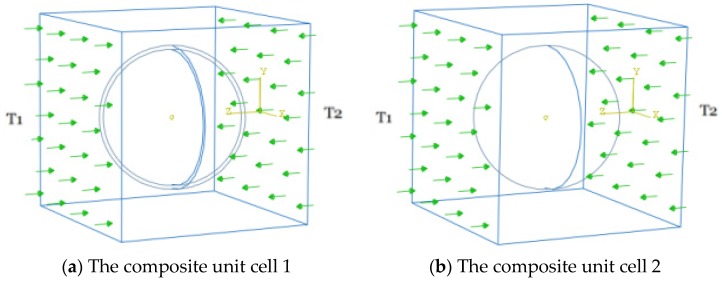
Heat transfer in the two composite systems under applied temperature constraints.

In order to determine the effective thermal conductivity of the two composite systems related to the HGM and its equivalent, two different constant temperature boundary conditions $T_{1}$ and $T_{2}$ ($T_{1}>T_{2}$) are applied on the opposite surfaces of the unit cell, i.e., the two surfaces perpendicular to the z axis, to make thermal energy flow through the unit cell from one surface to another, as shown in Figure 12. The remaining four surfaces are assumed to be insulated. Based on the constitutive formulation (5), the effective thermal conductivity of the composite system can be given by [42,43]

(7) $$k_{eff}\approx -\frac{{\tilde{q}}_{z}}{\nabla T_{z}}\approx \frac{{\tilde{q}}_{z}L}{T_{1}-T_{2}}$$

in which ${\tilde{q}}_{z}$ indicates the averaged heat flux component on the surface perpendicular to the z axis, i.e., the surface z=L with the constant temperature constraint $T_{2}$, and $\nabla T_{z}$ is the temperature gradient between the two opposite surfaces perpendicular to the *z* axis, which can be evaluated by $\nabla T_{z}=(T_{2}-T_{1})/L$.

In the following computation, we assume $T_{1}=30\;℃\;\left(303.15\;K\right)$ and $T_{2}=10\;℃\;\left(283.15\;K\right)$ to create the temperature difference between the opposite surfaces perpendicular to the *z* axis to drive the thermal energy to flow in the unit cell.

#### 4.2.4. Results and Discussion

**Figure 13:**
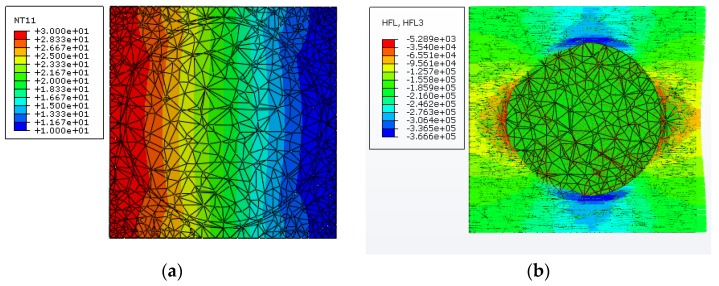
Distributions of (**a**) the temperature and (**b**) the heat flow $q_{z}$ along the *z* direction in the three-phase composite unit cell with 20% microsphere volume fraction.

To demonstrate the heat transfer behavior in the three-phase composite system, including the HGM filler and the matrix material, Figure 13 displays the variations of temperature and heat flux in the composite unit cell for a microsphere volume fraction of 20%, in which the length and direction of the arrow, respectively, indicate the strength and direction of the heat flow component $q_{z}$ along the *z* direction. It is clearly observed from Figure 13 that the temperature distribution in this three-phase composite unit cell is obviously nonlinear, which is caused by the presence of the hollow microsphere. Besides, it is seen that the route of heat transfer in the composite becomes longer when comparing to that in the pure matrix, owing to the presence of the spherical HGM. Moreover, the big difference of thermal conductivity of the solid material and the gas phase inside the HGM leads to most of heat energy to flow around the microsphere wall.

Making use of Equation (7), the effective thermal conductivity of this three-phase composite system can be evaluated for various microsphere volume fractions. It is found that the effective thermal conductivity of the composite significantly decreases with the increase of microsphere volume fraction. When compared to the pure matrix material with thermal conductivity 0.93 W/(mK), there are 11.4%, 22%, 31.9%, and 41.2% decreases of the effective thermal conductivity $k_{eff}$ when the microsphere volume fraction is equal to 10%, 20%, 30%, and 40%, respectively. This is mainly attributed to the hollow glass microsphere, which has lower thermal conductivity than the matrix material.

**Figure 14:**
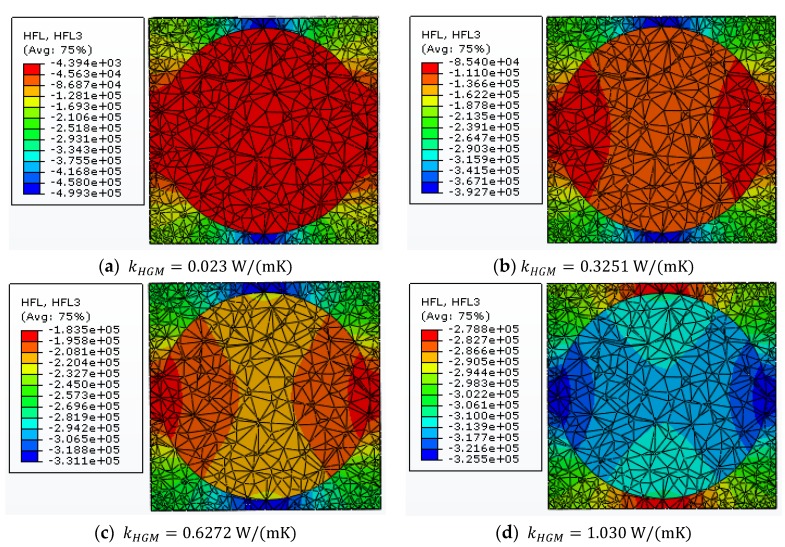
Variations of heat flux component $q_{z}$ along the *z* direction in the two-phase composite unit cell with 20% microsphere volume fraction.

Following the hollow feature of HGM, we find that the thermal conductivity of the HGM $k_{HGM}$ must be greater than 0.023 W/(mK) and less than 1.030 W/(mK), which, respectively, represents the thermal conductivities of the solid wall and the gas. Thus, for the two-phase composite system including the equivalent filler and the matrix material, it is assumed that the thermal conductivity of the equivalent homogeneous solid microsphere changes from 0.023 W/(mK) to 1.030 W/(mK). Figure 14 shows the variations of heat flux component $q_{z}$ along the *z* direction in the two-phase composite unit cell for different $k_{HGM}$. The filler volume fraction is 20%. It is found that the heat transfer will always follow the material with higher thermal conductivity and more heat energy goes through the equivalent solid microsphere with the increase of $k_{HGM}$.

**Figure 15:**
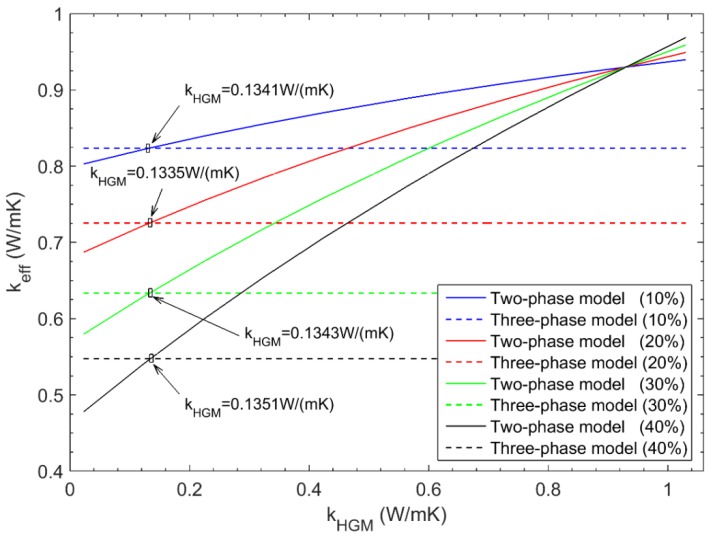
Results from the two-phase and three-phase composite systems for different microsphere volume fractions.

Figure 15 indicates the variation of the effective thermal conductivity of the two-phase composite system with respect to $k_{HGM}$ for various microsphere volume fractions. To obtain the thermal conductivity of the single HGM element, the equivalent results from the three-phase composite system are also plotted in Figure 15 for each microsphere volume fraction (see the straight lines in Figure 15). The intersection of the straight line from the three-phase composite system and the curved line from the two-phase composite system gives 0.1341 W/(mK), 0.1335 W/(mK), 0.1343 W/(mK), and 0.1351 W/(mK), respectively. As expected, the thermal conductivity $k_{HGM}$ of the HGM is not sensitive to the microsphere volume fraction to the unit cell, and the almost same result is obtained for different microsphere volume fractions. Theoretically, one can arbitrarily choose any microsphere volume fraction for the computation analysis. However, the strong interaction between the cell boundary and the microsphere may exist for large microsphere volume fraction, hence it is suggested to employ the relative small or medium microsphere volume fraction in the practical computation. Here, the average value 0.1343 W/(mK) is used as the approximated thermal conductivity of the single HGM.

Additionally, in order to investigate the independence of the predicted result to the choice of fictitious matrix material, it is assumed that the thermal conductivity of the fictitious matrix material changes to 0.53 W/(mK). The resulted thermal conductivity of the single HGM is 0.1332 W/(mK), under 20% filler volume fraction to the composite unit cell. This result is almost same to that for the matrix with thermal conductivity 0.93 W/(mK). Thus, it can be concluded that the final result is independent of the choice of fictitious matrix in the present method.

It should be noted that the experimental result of HGM powder (0.1014 W/(mK)) is obviously less than the computational result of the single HGM stacking element (0.1343 W/(mK)), as we expect. The main reason is that there are large numbers of voids between spherical particles in the actual HGM powder. Because the TPS method is a contact method, the existence of void decreases the heat transfer efficiency from the heating sensor to the powder and naturally leads to smaller experimental results. Therefore, in order to obtain the thermal conductivity of the HGM powder, the cubic and truncated octahedron HGM stacking elements displayed in Figure 8 should be introduced into the present computational model to represent actual distribution of HGMs in the powder as possible. Following the procedure mentioned above, the effective thermal conductivity of the cubic and truncated octahedron HGM stacking elements can be predicted as 0.06286 W/(mK) and 0.09652 W/(mK), respectively. As we expect that the truncated octahedron arrangement of HGMs produces larger thermal conductivity than the cubic arrangement, due to the difference of void volume fraction inside the element. Moreover, it is found that the effective thermal conductivity of the truncated octahedron HGM stacking element is very close to the experimental result (0.1014 W/(mK)) than that of the cubic HGM stacking element, so the hexagonal close-packing can better represent the real distribution of HGMs in the powder than the cubic close-packing. Moreover, the consistency of numerical and experimental predictions indicates that both the TPS measurement and the present numerical model can be used for determining the thermal conductivity of HGM powder. Besides, it is seen that both the cubic HGM stacking element and the truncated octahedron HGM stacking element lead to smaller thermal conductivity than the single HGM stacking element. This is reasonable because the external surrounding void is introduced for the cubic and truncated octahedron HGM stacking elements. More importantly, from the basic procedure described above, we find that the present computational model can be flexibly applied to determine the thermal conductivity of other complex-shaped fillers, without any difficulty.

## 5. Conclusions

Owing to its hollow structure and spherical shape, the overall thermal conductivity of tiny hollow glass microsphere is generally difficult to be measured or evaluated directly. In this study, the effective thermal conductivities of the single HGM and its powder are respectively investigated by the powerful TPS method and the developed three-dimensional two-step hierarchical computational method. The results obtained leads to the following conclusions: (1) The TPS method can only be used to experimentally measure the thermal conductivity of HGM powder, which can detect in-homogeneities in the HGM powder, such as the non-uniformity of particle size and the presence of air voids between neighboring HGMs. (2) The proposed two-step hierarchical computational model can be employed to effectively characterize the overall thermal conductivity of single HGM and its powder. (3) In the present computational scheme, the predicted results are independent of the choice of fictitious matrix material and the change of filler volume fraction to the composite cell. (4) The hexagonal close-packing can better represent the real distribution of HGMs in the powder than the cubic close-packing and the single microsphere. (5) From the computational procedure, it is found that the developed two-step computational method can be extended to determine the overall thermal conductivity of other complex-shaped heterogeneous fillers, without any difficulty.

## Figures and Tables

**Table 1:** Experimental results of the HGM powders.

Group	Heating Power (W)	Temperature Increase (K)	Thermal Conductivity (W/(mK))
A	0.061	282.78	0.0981
	0.071	283.79	0.1005
	0.081	285.29	0.1014
B	0.061	283.25	0.0877
	0.071	284.76	0.0969
	0.081	284.39	0.1054
C	0.061	281.81	0.1083
	0.071	284.11	0.0943
	0.081	284.69	0.1115
D	0.061	282.73	0.0952
	0.071	284.30	0.1013
	0.081	285.40	0.1025

**Table 2:** Geometrical and physical properties of HGM for simulation.

Particle Size	Value
Average outer diameter *D =* 2R (μm)	58.64
wall thickness *t* (μm)	1.6
Thermal conductivity	
Thermal conductivity of the gas $k_{g}$ (W/(mK))	0.023 [38]
Thermal conductivity of the solid wall $k_{w}$ (W/(mK))	1.03
Thermal conductivity of the matrix $k_{c}$ (W/(mK))	0.93
